# A comparison of piezoelectric surgery and conventional techniques in the enucleation of cysts and tumors in the jaws: a systematic review and meta-analysis

**DOI:** 10.4317/medoral.26799

**Published:** 2025-03-23

**Authors:** Lucía Suárez-Pérez, Mariela Peralta-Mamani, Rocío Trinidad Velázquez-Cayón

**Affiliations:** 1 Professor. Department of Dentistry, Faculty of Health Sciences, University Fernando Pessoa Canarias; 2 Hospital for Rehabilitation of Craniofacial Anomalies, University of São Paulo, University of São Paulo; 3 Professor. Paulista Central-West College-FACOP, São Paulo, Brazil

## Abstract

**Background:**

Despite the comprehensive classifications provided by the WHO, the most common lesions include radicular cysts, dentigerous cysts, odontogenic keratocysts, ameloblastomas, and odontomas. The piezoelectric technique has shown effectiveness in removing intraosseous pathologies by relying on ultrasonic microvibrations, which help preserve soft and vascular tissues. Precision in manipulating intraosseous pathology can impact the prognosis and improve the surgical procedure by controlling hemorrhage and promoting microscopic benefits. While previous research has compared the advantages of piezoelectric surgery and rotational methods, a systematic review is needed to consolidate the available information on this specific clinical issue.

**Material and Methods:**

A search strategy was developed with de PRISMA statement. PubMed, Web of Science, Scopus, and Embase electronic databases were searched. The bibliographic search was conducted in December 2023. The methodological quality of the studies followed the Joanna Briggs Institute (JBI) critical evaluation tool for randomized clinical trials.

**Results:**

The final sample comprised 5 clinical trials, involving 231 cysts and 120 tumors in the experimental group. The mean age of participants was 30.6 years, with 196 men and 141 women included in the study. However, conventional surgery is faster than piezosurgery, both techniques exhibited similarities in epithelial perforation, soft tissue damage, edema, postoperative infections, and occurrences of paresthesia. Regarding recurrence, no statistically significant difference was observed between the two techniques (*p-value*=0.339; 95% confidence interval, -0.093-0.270).

**Conclusions:**

The surgical removal of benign odontogenic cysts and tumors in the jaws using piezosurgery yielded slight intraoperative and postoperative advantages compared to conventional rotary surgery, except for the duration of surgical procedures. It shows reduced intraoperative hemorrhage and postoperative pain but similar outcomes in other variables. The results should be interpreted with caution, more studies are needed to obtain a more robust result.

** Key words:**Piezosurgery, maxillofacial surgery, radicular cyst, dentigerous cyst, ameloblastoma, odontoma, systematic review.

## Introduction

Benign lesions affecting the jaws, including cysts and odontogenic tumors, exhibit diverse origins, being inflammation the most prevalent cause (1,2). However, these lesions can also originate from embryonic remnants (2,3). Cysts, notably more common than tumors, encompass the radicular cyst as the primary type, followed by dentigerous and odontogenic keratocysts. In the realm of tumors, intraosseous ameloblastoma ranks highest in frequency, succeeded by odontoma (2). The precise definition of a cyst as a "connective-epithelial pocket lined on the inside by epithelium and covered on the outside by connective tissue, containing a liquid or semi-liquid content," and of a tumor as a "solid mass, not necessarily neoplastic," establishes the foundation for pathological classification based on the origin of the epithelial lining and the origin of formation of ectodermal, mesenchymal types, or both (3,4). To ensure uniform identification across disciplines, the World Health Organization (WHO) has issued multiple classifications of these injuries over the years (5-6).

Factors such as size, type, nature, location, etiology, and the patient's age play a crucial role in determining the appropriate treatment (7,8). Therapeutic options for cysts include cystectomy, cystotomy, and decompression, each with specificities and advantages. Conversely, the treatment of tumors often involves enucleation or resection, and potential recurrences may necessitate adjuvant treatments (9-12).

Piezoelectric surgery, a modern and precise technique, has emerged as an effective and safe alternative to address such injuries, enabling selective sectioning of hard tissues while preserving surrounding soft tissues (13-15).

Technological advances in oral surgery have benefited from this alternative, as piezoelectric surgery minimizes the risks of neurological injuries during osteotomies close to highly innervated tissues (16). Unlike traditional tools, piezoelectric surgery preserves soft tissues by executing selective osteotomies in hard tissues, thus avoiding collateral damage (17). Additionally, it enhances visibility by reducing bleeding in the operating field, overcoming the limitations of conventional surgical techniques (18). Studies indicate that bones treated with traditional burs may not be suiTable for grafting due to the lack of osteocytes, underscoring the importance of selecting appropriate tools. The piezoelectric technique demonstrates effectiveness in the removal of intraosseous pathologies, with its mechanism of action relying on ultrasonic microvibrations, thereby preserving soft and vascular tissues. Precision in manipulating intraosseous pathology influences the prognosis and enhances the surgical procedure by controlling hemorrhage and promoting microscopic benefits (19).

Despite the comprehensive classifications provided by the WHO, the most common lesions are radicular cysts, dentigerous cysts, odontogenic keratocysts, ameloblastomas and odontomas (20). While prior research has compared the advantages between piezoelectric surgery and rotational methods, a systematic review is warranted to synthesize the available information on this specific clinical issue.

Considering the above, the primary objective of this systematic review is to investigate whether piezoelectric surgery is effective in treating benign odontogenic cysts and tumors of the jaws in healthy patients or those with mild controlled pathologies. To this end, the following research question was posed: Does piezoelectric surgery offer advantages over conventional rotational surgeries in the treatment of tumors and odontogenic cysts?

## Material and Methods

- Protocol recording

The PRISMA (Preferred Reporting Items for Systematic Reviews and Meta-Analyses) guidelines were adhered to in guiding this review (21). The study protocol was registered in the PROSPERO database with the identification number CRD42023493011.

- Focused question

For cases of radicular cyst, residual cyst, dentigerous cyst, odontogenic keratocyst, odontoma, and ameloblastoma, what are the results of enucleation with piezoelectric instruments compared to rotary instruments during intraoperative, postoperative, and long-term outcomes?

- Eligibility criteria

The PICO strategy was employed to frame the eligibility criteria. Included studies needed to address all components of PICO, with the population comprising patients diagnosed with radicular cyst, residual cyst, dentigerous cyst, odontogenic keratocyst, odontoma, and ameloblastoma. The intervention considered was enucleation with piezosurgery, with conventional rotary instruments serving as the control. Outcomes of interest included clinical parameters during the intraoperative phase (bleeding, visibility, epithelial wall perforation, ease of surgery), postoperative phase (edema, pain, paresthesia), and long-term recurrence rate.

Exclusion criteria comprised studies involving patients on antiplatelet or anticoagulant treatments, syndromic patients, or those with moderate or severe systemic diseases. Additionally, bibliographic reviews, letters to the editor, comments, editorials, cross-sectional studies, and observational or descriptive studies were excluded.

- Information sources and search strategy

The bibliographic search, conducted in December 2023, encompassed databases such as PubMed, Web of Science, Scopus, and Embase. Employing Medical Subject Headings (MeSH) in combination with Boolean operators "AND" and "OR," a tailored search strategy was adapted for each database. The search string covered terms related to both piezoelectric surgery and a spectrum of cysts and tumors: (Piezosurgery OR Piezo-surgery OR piezoelectric OR Piezo-electric OR “Piezoelectric bone surgery” OR “piezoelectric surgery” OR “Ultrasonic surgery” OR “Ultrasonic bone curette”) AND (“Bone cysts” OR “Jaw cyst” OR “Jaw cysts” OR “Mandibular cyst” OR “Radicular Cyst” OR “Apical Periodontal Cyst” OR “Apical Periodontal Cysts” OR “Cyst, Apical Periodontal” OR “Cyst, Periapical” OR “Cyst, Radicular” OR “Cysts, Apical Periodontal” OR “Cysts, Periapical” OR “Cysts, Radicular” OR “Periapical Cyst” OR “Periapical Cysts” OR “Periodontal Cyst, Apical” OR “Periodontal Cysts, Apical” OR “Radicular Cysts” OR “Dentigerous Cyst” OR “Cyst, Dentigerous” OR “Cysts, Dentigerous” OR “Dentigerous Cysts” OR Keratocysts OR Keratocyst OR “Odontogenic keratocyst” OR “Odontogenic keratocysts” OR OKC OR OKCs OR “Keratocystic odontogenic tumours” OR “Keratocystic odontogenic tumour” OR KCOT OR KCOTs OR Odontoma OR Odontomas OR “Odontoma, Compound” OR “Compound Odontoma” OR “Compound Odontomas” OR “Odontomas, Compound” OR “Complex odontoma” OR “Compound-complex odontoma” OR Ameloblastoma OR Ameloblastomas OR “Unicystic ameloblastoma” OR “Peripheral ameloblastoma” OR “Multicystic ameloblastoma”).

- Selection process

After database searches, duplicates were excluded using the Zotero bibliographic reference manager (version 6.0.26). Initial screening involved reviewing titles and abstracts, with subsequent full-text reading for final selection. Inclusion and exclusion criteria were consistently applied. Primary study selection involved two independent reviewers who carried out an initial screening based on the review of titles and abstracts (RVC and LSP), with a third reviewer (MPM) resolving discrepancies. The Kappa concordance test yielded a coefficient of 0.72, indicating considerable agreement and reinforcing the reliability of the study selection process.

- Data collection processing and data items

Data collection was performed by two reviewers (RVC and LSP), focusing on clinical trial results comparing piezoelectric surgery to conventional surgery in the enucleation of maxillary cysts and tumors. Criteria ensured uniformity and relevance, with a detailed analysis at intraoperative, postoperative, and long-term phases. Collected data included patient demographics, surgery-related details, and outcomes. Intraoperative data covered surgery time, bleeding, visibility, and complexity of cyst and tumor removal. Postoperative information encompassed pain and edema, while recurrence rates were assessed for the long-term phase. This meticulous data collection strategy facilitated a comprehensive and nuanced analysis of the effectiveness of piezoelectric surgery compared to conventional surgery in maxillary cyst and tumor enucleation, with specific findings detailed in each category within the study results.

- Risk of bias assessment

The methodological quality of the studies followed the Joanna Briggs Institute (JBI) critical evaluation tool for randomized clinical trials. Each study was independently assessed, and the overall methodological quality was categorized as having a high, moderate, or low risk of bias. The following critical evaluation criteria were considered, with responses categorized as 'yes,' 'no,' or 'not clear':

1. Proper random assignment: Evaluation ensured the description of an appropriate randomization process, ensuring equiTable distribution of participants across treatment groups.

2. Assignment hiding: It assessed whether the randomization assignment process was sufficiently concealed to minimize the risk of bias.

3. Blinding of participants and staff: Examination of whether participants and involved staff were adequately blinded to avoid bias in treatment implementation.

4. Blinding of outcome: Assessment of whether outcome evaluators were blinded in the treatment group to minimize bias in outcome measurement.

5. Handling incomplete data: Examination of data completeness, considering participant loss and how missing data were addressed.

6. Selection of reported results: Evaluation of the consistency between reported results and those pre-specified in the study protocol.

Other sources of bias: Examination of possible sources of bias not covered by previous criteria.

- Effect Measures

The comparative evaluation between piezoelectric surgery and conventional surgery for enucleation of maxillary cysts and tumors involved various effect measures. The Visual Analogue Scale (VAS) was used to assess cutting, visibility, ease of intervention, and ease of cystic wall removal, with scores ranging from 0 to 10. Operation time, recurrences, bone graft infection, and graft breakage were reported in absolute values. Pain was reported using the VAS scale (0: absence of pain, 10: maximum painful sensation). Edema was measured using a cephalometer (distance between the skin and a permanent marker before surgery) after 24 h, 48 h, 72 h, and one week. Edema and other activities such as lockjaw, chewing, talking, and sleeping were evaluated on a five-point Likert-type scale ranging from 1 to 5 (1 = not at all, 2 = very little, 3 = average, 4 = quite a bit, and 5 = a lot). Paresthesia was reported as absolute values (number of cases), and intraoperative hemorrhage was expressed using the VAS (0: without hemorrhage control, 1: mild intermittent hemorrhage, 2: complete hemorrhage control).

- Synthesis methods

A narrative synthesis of the selected studies was conducted, comparing results descriptively and analytically across the intraoperative, postoperative, and long-term phases. Intraoperative variables included time, visibility, hemorrhage, cystic epithelial perforation, intervention difficulty, and soft tissue damage. Postoperative differences between techniques regarding pain, edema, hemorrhage, paresthesia, and infection were reported. Long-term success was assessed by comparing recurrence rates between piezoelectric and conventional surgery.

Quantitative analysis was performed through meta-analysis using OpenMeta[Analyst]® software, employing a random-effect model (22) with a 95% confidence interval and a 5% significance level. Sensitivity analysis was conducted in cases of high heterogeneity.

- Certainty assessment

Certainty of evidence was explored using the GRADE tool (Grading of Recommendations, Assessment, Development and Evaluation) (23), which classifies the level of scientific evidence into categories. Accordingly, randomized clinical trials are associated with a high level of scientific rigor, whereas the converse holds true for observational trials. Hence, all studies incorporated into the review were randomized clinical trials.

## Results

- Study selection

**Figure 1:**
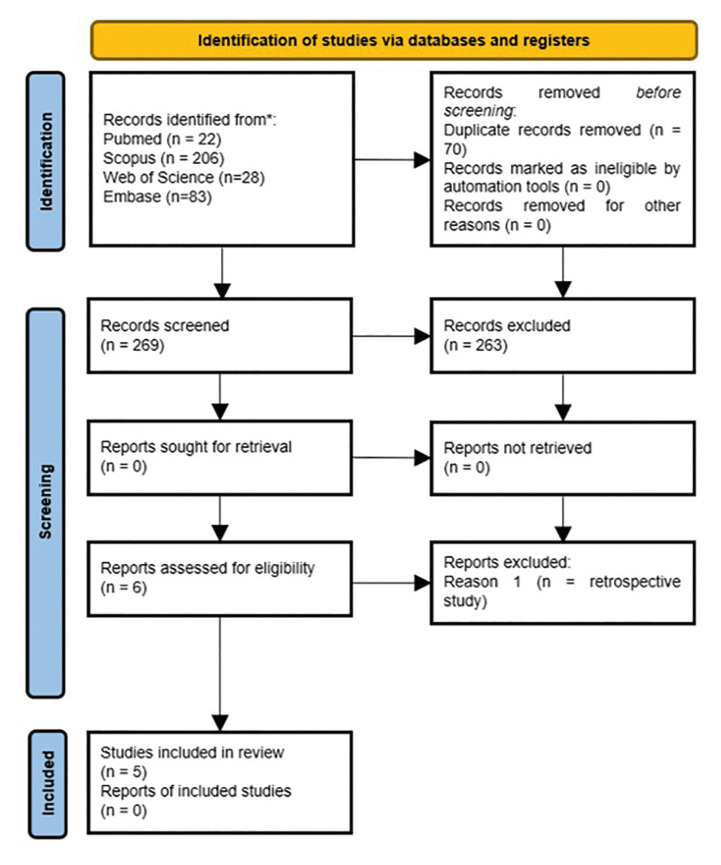
PRISMA Flowchart detailing the study selection process.

The initial database search, conducted without filters, returned 339 studies: 83 from Embase, 22 from PubMed, 206 from Scopus, and 28 from Web of Science. Following the assessment of titles and abstracts, six studies were initially chosen, with one exclusion post full-text examination due to its retrospective nature. Consequently, the final systematic review comprised five studies. Inter-examiner agreement, evaluated using the Kappa test, resulted in a substantial coefficient of 0.72, affirming a reliable study selection process. Fig. 1 illustrates the flowchart detailing the study selection process from the initial search to the final inclusion in this systematic review.

- Study characteristics

This systematic review exclusively focuses on randomized clinical trials, featuring a mean participant age of 30.6 years across all trials. The participation comprised 196 men and 141 women, with 182 lesions enucleated using piezoelectric surgery and 169 lesions using conventional surgery. The studies spanned from 2012 to 2021, with one at the Postgraduate Institute of Dental Sciences, Rohtak, Haryana, India (24), with two conducted in Turkey (25,26), two in Italy (Catania and Naples) (27,28). Among the lesions, three studies evaluated 117 radicular cysts (24-26), with Yaman *et al*. (25) additionally addressing 12 dentigerous cysts, 13 residual cysts, and 9 odontogenic keratocysts. Pappalardo *et al*. (27) performed surgery on 80 cysts without specifying the type, mentioning initial endodontic treatment for radicular cyst cases. Marra *et al*. (28) focused on the enucleation of 120 odontomas. Refer to Table 1 for detailed information.

- Risk of bias

The evaluation of the risk of bias revealed that most studies demonstrated moderate to high methodological quality. Two studies presented clear random assignment, allocation concealment, and blinding (24,27); however, elements such as the similarity of treatment between groups were not specified (24). Three studies demonstrated moderate methodological quality. One study (25) showed clarity in random assignment and handling of incomplete data but lacked definition in group assignment concealment and the similarity between treatment groups. Another study (26) had undefined aspects, such as group assignment concealment and participant blinding during the intervention. A third study (28) also lacked clarity in random assignment and blinding of both participants and operators during the intervention. Table 2 provides a detailed overview of the methodological quality for each study.

- Results of individual studies

- Intraoperative Variables

Two studies assessed intraoperative hemorrhage. Bharathi *et al*. (24) observed greater bleeding with conventional surgery, as did Kocyigit *et al*. (26), who reported three uncontrolled cases of intraoperative hemorrhage in the control group, unlike the group undergoing piezoelectric surgery, in which none was recorded.

Only Yaman *et al*. (25) analyzed the ease of cutting. The authors found that piezoelectric surgery facilitated the cutting of hard tissues compared to conventional surgery with rotary instruments.

Two studies evaluated cyst epithelial perforation. Yaman *et al*. (25) did not show significant differences between groups. Kocyigit *et al*. (26) presented favorable results for piezoelectric surgery since there were no cases of perforation of the epithelial wall of the cyst, unlike the control group, with five cases.

Two studies evaluated the difficulty of intervention. Yaman *et al*. (25) showed greater ease of intervention in groups treated with piezoelectric surgery according to the VAS, as did Kocyigit *et al*. (26), who described five cases of intervention difficulty in the control group, while in the piezoelectric surgery group there were no difficulties.

Only Yaman *et al*. (25) evaluated visibility, showing favorable results in groups treated with piezoelectric surgery according to the VAS.

Yaman *et al*. (25) evaluated complications and described two cases of insert breakage with piezoelectric surgery that resulted in an increase in intervention time.

Two studies evaluated the variable referring to soft tissue damage. Kocyigit *et al*. (26) reported no cases of soft tissue damage in either group, as did Bharathi *et al*. (24), who found no significant differences between groups.

Three studies evaluated time. Yaman *et al*. (25) found significant differences between groups, with longer times in complicated surgeries using piezoelectric surgery. The results of the trials in Kocyigit *et al*. (26) showed that the piezoelectric surgery group exceeded the estimated intervention time in 100% of cases, unlike the control group, which did not exceed it in any case. Marra *et al*. (28) showed an increase in intervention time in the piezoelectric surgery group of an average of 18 minutes, unlike the control group, which averaged 12 minutes. Refer to Table 3 for detailed information.

- Postoperative Variables

Only Kocyigit *et al*. (26) studied postoperative hemorrhage and found that it was completely controlled with piezoelectric surgery, while in the control group there were two cases of postoperative bleeding.

Three studies evaluated postoperative pain. Pappalardo *et al*. (27) showed a significant reduction in pain in the group treated with piezoelectric surgery in the first seven days. Bharathi *et al*. (24) also reported less pain in the piezoelectric surgery group during the first two days, requiring less analgesics than the rotational group. Marra *et al*. (28) also reported less pain in the piezoelectric surgery group.

Two studies evaluated edema. Pappalardo *et al*. (27) showed a significant reduction in edemas during the first days in the group treated with piezoelectric surgery. Marra *et al*. (28) found similar results.

Yaman *et al*. (25) collected information regarding postoperative infections and did not find significant differences between both techniques since there were two cases of infection in each group.

Only Pappalardo *et al*. (27) studied paresthesia and showed beneficial results in the group treated with piezoelectric surgery, which did not present any cases, unlike the control group, in which there were two cases. Refer to Table 4 for detailed information.

- Long-term variables

In terms of long-term variables, two studies analyzed recurrences. Yaman *et al*. (25) showed no differences between both groups in relation to recurrences, while Kocyigit *et al*. (26) showed favorable results for piezoelectric surgery at six months, with no cases of recurrence, unlike the control group, in which there were two cases of recurrence. Refer to Table 4 for detailed information.

- Results of syntheses

**Figure 2:**
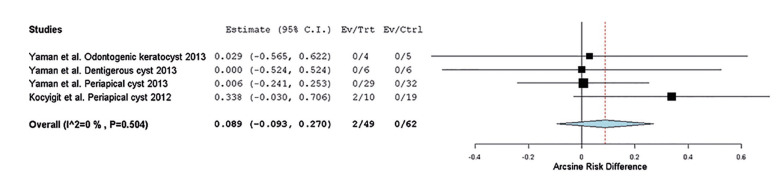
Forest plot illustrating the recurrence of dentigerous cyst, odontogenic keratocyst, and periapical cyst.

The meta-analysis of recurrence encompassed two studies with quantitative data (25,26). The analysis considered dentigerous cyst (one study), odontogenic keratocyst (one study), and periapical cyst (two studies). The results did not reveal a statistically significant difference between the group treated with conventional surgery and the group treated with piezoelectric surgery (*p-value* = 0.339; estimate 0.089; 95% confidence interval, -0.093-0.270; heterogeneity: Q value 2.345, I2 0%; Tau2 0.000; *p-value* 0.504) (Fig. 2).

**Figure 3:**
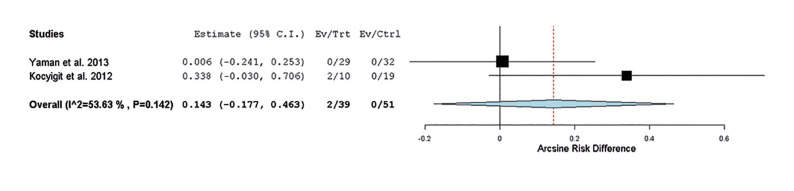
Forest plot depicting the recurrence of dentigerous cyst, odontogenic keratocyst, and periapical cyst following sensitivity analysis.

In a subgroup analysis focusing solely on studies evaluating periapical cyst recurrence, the outcome remained unchanged. No statistically significant difference between the groups emerged (*p-value* = 0.382; estimate 0.143; 95% confidence interval, -0.177-0.463; heterogeneity: Q value 2.157, I2 53.634%; Tau2 0.030; *p-value* 0.142) (Fig. 3).

## Discussion

The primary effects of intervention using piezoelectric surgery exhibited favorable outcomes, manifested through reduced intraoperative hemorrhage (24,26), pain, and inflammation. However, the duration of procedures was shorter in the group treated with conventional rotational surgery (25,26,28). Other beneficial outcomes of interventions employing piezoelectric surgery were observed concerning visibility, perforation of the epithelial wall, paresthesia, and recurrences (24-28).

The meta-analysis was conducted on data from trials providing recurrence frequency information, specifically from Kocyigit *et al*. (26), Yaman *et al*. (25). The analysis included one study on dentigerous cyst (25), one on odontogenic keratocyst (25), and two on periapical cyst (25,26). The results showed no statistically significant differences between the group treated with conventional surgery and the group treated with piezosurgery (*p*=0.339). Subgroup analysis focusing solely on studies evaluating the recurrence of periapical cysts yielded similar results (*p*=0.382). Postoperative pain primarily results from tissue trauma (29). Three studies within this systematic review evaluated pain one week after surgery, while Marra *et al*. (28) monitored pain only within the initial 72 hours. All studies reported beneficial outcomes for piezoelectric surgery, indicating a reduction in pain compared to the control group. These findings align with studies demonstrating that piezoelectric surgery reduces the release of inflammatory mediators compared to conventional rotational surgery (30). Significant differences in pain during the first seven days were evident in the study of Pappalardo *et al*. (27), whereas Bharati *et al*. (24), observed significant differences only on the first and second postoperative days. Similarly, Bharati *et al*. (24) documented significant differences in analgesic consumption in the first three days post-surgery indicating a reduced need for analgesia in the piezoelectric surgery group. Further high-quality clinical trials, incorporating standardized guidelines for anti-inflammatory medications and pain assessment tools, are necessary for more conclusive results. In comparison to conventional rotary instruments, piezoelectric surgery inflicts less damage to bone tissues, fostering a better blood supply and resulting in a lower incidence of postoperative inflammation (29). This hypothesis may partially explain the study results. Two trials assessed postoperative facial edema, revealing significant differences with a decrease in edema using the piezoelectric technique compared to conventional rotational surgery. Factors such as age, flap design, or medication could influence facial swelling outcomes. Consequently, further studies are warranted, considering consistent references, and standardizing the anti-inflammatory regimen to evaluate facial edema and obtain conclusive results. Some studies have concluded that piezoelectric surgery requires a longer operation time compared to the use of high-speed instruments, which could potentially cause discomfort to the patient (31). Four trials compared operation times between both groups. In Bharati *et al*. (24), no significant differences were noted between the two types of interventions. Yaman *et al*. (25), however, reported significantly longer operation times in the piezoelectric surgery group compared to the conventional surgery group for both simple and complex cases. These findings are supported by Marra *et al*. (28), who also found significant differences between both groups. Likewise, Kocyigit *et al*. (26) indicated that in 100% of patients treated with piezoelectric surgery, the intervention time exceeded the allotted time, whereas no such exceedance occurred in the control group. Most aggregated results suggest that piezoelectric surgery demands more operation time.

Piezoelectric surgery exhibits selectivity towards bone, safeguarding soft tissues such as blood vessels and nerves (32). The precision of cutting in piezoelectric surgery is attributed to ultrasonic microvibrations within the frequency range of 25-30 kHz, enabling the cutting of mineralized tissue while sparing soft tissues affected only by frequencies higher than 50 kHz (33). In the clinical trial reviewed by Kocyigyt *et al*. (26), the study explored the perforation of the epithelial wall of the cyst during interventions using both conventional and piezoelectric techniques. The results favored piezoelectric surgery, as it revealed no instances of perforation of the epithelial wall of the cyst, unlike the rotational group where the integrity of the epithelial wall was compromised in five cases. The study also analyzed damage to surrounding soft tissues near the surgical area, revealing no alterations in either group. Of all trials included in the review, only Kocyigyt *et al*. (26) provided results on perforation of the epithelial wall and damage to soft tissues, suggesting the incorporation of these variables in future studies. These findings align with previous studies highlighting perforation of the sinus membrane as a frequent complication during sinus lift procedures (14-56%), often associated with difficulties in using manual tools. Piezoelectric surgery devices, with inserts offering a variety of angles, facilitate membrane elevation with greater ease and efficiency (15).

In 2008, Schaeren *et al*. (13) investigated cutting selectivity, evaluating nerve tissue damage through ultrasonic surgery during osteotomies. They asserted that even direct exposure to a nerve with a piezoelectric device did not cause damage. In the current systematic review, only one trial (27) provided information on postoperative neurological pathologies. Patients treated with piezoelectric devices did not exhibit postoperative neurological alterations, unlike the conventional rotary group, which experienced two cases of paresthesia. However, in the retrospective study by Troiano *et al*. (24), despite the difference in incidence between both groups (three cases in the rotary surgery group and one case in the piezoelectric surgery group), no significant differences were observed between the two techniques. According to Schlee *et al*. (34), the improved visibility offered by piezoelectric surgery during interventions, as compared to rotational surgery, is attributed to the cavitation effect created by an irrigation solution and an oscillating tip. This effect allows blood removal through washing. Yaman *et al*. (25) corroborated this information in their trial using the Visual Analog Scale (VAS), indicating that the piezoelectric technique provided superior visibility to surgeons during interventions. Bharathi *et al*. (24) also noted improved hemorrhage control in the piezoelectric surgery group. The benefits of piezoelectric surgery regarding intraoperative hemorrhage control were further supported by the results of the clinical trial by Kocyigit *et al*. (26) where no cases of intraoperative hemorrhage occurred with piezoelectric surgery, unlike rotational surgery, which presented three cases. However, it is important to note that the two studies evaluating intraoperative hemorrhage used different scales, necessitating further clinical trials for unified information on this variable. Kocyigit *et al*. (26) also investigated bleeding in the days following surgery, revealing benefits associated with piezoelectric surgery.

Kocyigit *et al*. (26) demonstrated that the conventional rotary technique presented manipulation complexity in five cases, while interventions with piezoelectric surgery showed no instances of difficulty during the procedure. However, in the clinical trial of Yaman *et al*. (25), there were no significant differences between the techniques according to the Visual Analog Scale (VAS), which is utilized to evaluate the ease of intervention for various operations.

Piezoelectric surgery minimizes hemorrhage, ensuring a wide field of vision and facilitating the detection of cystic remains (34). The results of Kocyigit *et al*. (26) confirm this theory, as recurrences in the piezoelectric surgery group were 0%, compared to two cases in the control group over six months. In a retrospective study, Troiano *et al*. (14) analyzed recurrence five years after ameloblastoma interventions, favoring the group treated with piezoelectric surgery with a recurrence rate of 7.1%, while the conventional surgery group exhibited a recurrence of 30.7%. However, the trial by Yaman *et al*. (25) showed no cases of recurrence at 57 months in either group. The meta-analysis did not reveal a statistically significant difference between the group treated with conventional surgery and the group treated with piezoelectric surgery (*p-value*=0.339; estimate 0.089; 95% confidence interval, -0.093-0.270; heterogeneity: Q value 2.345, I2 0%; Tau2 0.000; *p-value* 0.504) (Fig. 2). Likewise, considering only studies evaluating periapical cyst recurrence, the results were consistent (*p-value*=0.382; estimate 0.143; 95% confidence interval, -0.177-0.463; heterogeneity: Q value 2.157, I2 53.634%; Tau2 0.030; *p-value* 0.142) (Fig. 3). This thorough analysis suggests that the choice between conventional surgery and piezosurgery does not significantly impact cyst recurrence. Despite these consistent findings, further studies are needed to determine long-term success rates, considering the multifactorial nature of intraosseous pathology influenced by geographical, genetic, or age-related factors. Approaching the topic from different perspectives and considering all factors that increase the risk of bias is essential.

The results of this systematic review indicate that only the study by Yaman *et al*. (25) collected information regarding postoperative infections, showing no significant differences between both techniques, with two cases of infection in each group. Further studies are needed to standardize antibiotic regimens recommended for surgical intervention of benign odontogenic cysts and tumors of jaws to study the incidence of perioperative infections. However, of the two trials studying postoperative paresthesia, only one showed beneficial results for the group treated using piezoelectric surgery.

Liu *et al*. (35) conducted a systematic review and meta-analysis, which included 5 eligible RCTs with a total of 402 patients, to assess the differences between the use of piezosurgery and conventional rotary instruments in third molar surgery. They found that pain scores were lower at 6 or 7 days post-surgery, and swelling scores were reduced at 7 days after the procedure. Additionally, mouth opening was significantly lower in the piezosurgery group at 1 day post-surgery.

In connection with the sinus lift technique, the osteotomy and sinus membrane elevation were performed either with piezosurgery tips or rotative diamond burs and manual membrane elevators. In clinical conducted, authors such as Delilbasi *et al*., Martins *et al*., Bensaha (36-38) found results indicating both a lower rate of membrane rupture and fewer intraoperative and postoperative symptoms. However, more evidence is needed to support its protocolization for routine use in daily clinical practice.

In oral surgery, we must also take into account piezosurgery, which allows for accurate and safe performance of osteotomies for alveolar bone crest expansion and dental implant removal. This technique provides excellent clinical and biological results, particularly in terms of osteocyte viability (38,39).

One of the primary limitations of this systematic review is the scarcity of comparative clinical trials between both techniques. Furthermore, each trial used different piezoelectric devices, and different pathological entities were compared, increasing the risk of bias. Another limitation was the diversity of variables studied in each trial and the different measurement tools used in each of them. Finally, the lack of information regarding the methodology employed in the trials resulted in an average study quality during the critical reading phase. Further randomized clinical studies should evaluate the effects of piezoelectric surgery compared with conventional surgery in various odontogenic cysts and tumors, considering more variables such as intraoperative hemorrhage, pain, inflammation, visibility, difficulty, postoperative bleeding, and recurrence. Standardizing factors such as anti-inflammatory and antibiotic dosage regimens, considering age, size of the intraosseous lesion, and demographic factors, may impact the results.

It is crucial to highlight the clinical relevance of both the research question and the conclusions of this review.

The utilization of piezoelectric surgery for the surgical removal of cysts and benign tumors from the jaws emerges as a viable alternative for enucleating these lesions. Piezosurgery demonstrates slightly superior efficacy over conventional surgery in managing intraoperative hemorrhage, postoperative pain, and intervention difficulty. Noteworthy advantages include enhanced precision in cutting hard tissues, improved visibility during surgery, and reduced postoperative bleeding.

Conversely, rotary surgery exhibits higher operational speed when compared to piezoelectric surgery. Both techniques yield comparable outcomes concerning epithelial perforation of the cyst, soft tissue damage, edema, postoperative infections, and paresthesia. Notably, in the assessment of recurrences, no statistically significant differences between the two methods are observed, indicating that piezoelectric surgery offers no advantage over the conventional approach regarding the recurrence rate.

Acknowledging the limitations inherent in this systematic review, further research will contribute to a more comprehensive understanding of the clinical implications and outcomes associated with these surgical modalities.

## Figures and Tables

**Table 1:** Study characteristics.

Authors	Groups	Oral lesions	Age	Gender	Post-surgery recommendations /medication
Bharathi et al., 2021 (24)	Piezosurgery: 20 cysts Convencional: 20 cysts	40 radicular cysts	Piezosurgery group: Range: 18-44 Media: 27,85 Convencional group: Range: 16-51 Media: 26,15	Women=21 Men=19	No antibiotic prescribed Ibuprofen 400 mg Chlorhexidine digluconate 0.2% mouthwash (2/day for 1 week)
Yaman et al., 2013 (25)	Piezosurgery: 43 cysts Convencional: 39 cysts	48 radicular cysts 12 dentigerous cysts 13 residual cysts 9 odontogenic keratocysts	Range: 9-64 Media: 35,3 ± 13,5	Women=25 Men=43	Antibiotic prophylaxis: amoxicillin/clavulanic acid 1g/ or clindamycin 150 mg Paracetamol 500 mg or ibuprofen 400 mg Chlorhexidine digluconate 0.2% mouthwash (3/day for 1 week) Local cold for the first 12 hours after surgery
Kocyigit et al., 2012 (26)	Piezosurgery: 19 cysts Convencional: 10 cysts	29 radicular cysts	Range: 13-64 Media: 29,3	Women=13 Men=16	Antibiotic prophylaxis NSAIDs (non-steroidal anti-inflammatory drugs) Antiseptic mouthwash for (5 days after surgery
Pappalardo et al., 2013 (27)	Piezosurgery: 40 cysts Convencional: 40 cysts	80 radicular cysts	Range: 21-67 Media: 43,2	Women=45 Men=35	Antibiotic prophylaxis: amoxicillin/clavulanic acid 1g Ibuprofen 800 mg 3 days Chlorhexidine digluconate mouthwash
Marra, et al., 2020 (28)	Piezosurgery: 60 odontomas Convencional: 60 odontomas	120 compound odontomas	Rango: 19-25 Media: 22	Women=37 Men=83	-

**Table 2:** Assessment of included randomized controlled trials using Joanna Briggs Institute (JBI) critical appraisal.

JBI critical appraisal tool for randomized controlled trials	Bharathi et al. (24)	Yaman et al. (25)	Kocyigit et al. (26)	Pappalardo et al. (27)	Marra et al. (28)
Was true randomization used for assignment of participants to treatment groups?	YES	YES	YES	YES	YES
Was allocation to treatment groups concealed?	YES	NC	NC	YES	NC
Were treatment groups similar at the baseline?	YES	YES	NC	YES	YES
Were participants blind to treatment assignment?	YES	NC	NC	YES	NC
Were those delivering the treatment blind to treatment assignment?	NO	NC	NC	NO	NC
Were treatment groups treated identically other than the intervention of interest?	NC	NC	YES	YES	YES
Were outcome assessors blind to treatment assignment?	YES	YES	YES	YES	YES
Were outcomes measured in the same way for treatment groups?	YES	YES	YES	YES	YES
Were outcomes measured in a reliable way?	YES	YES	YES	NC	YES
Was follow-up complete and, if not, were differences between groups in terms of their follow-up adequately described and analyzed?	YES	YES	YES	YES	YES
Were participants analyzed in the groups to which they were randomized?	YES	YES	YES	YES	YES
Was appropriate statistical analysis used?	YES	YES	YES	YES	NC
Was the trial design appropriate and any deviations from the standard RCT design (individual randomization, parallel groups) accounted for in the conduct and analysis of the trial?	YES	YES	NC	YES	YES
Total quality assessment score for each study	84,6% (High)	69,2% (Moderate)	53,8% (Moderate)	84,6% (High)	61,5% (Moderate)

NC: not clear; §(High quality= 80-100%, medium quality= 50-79%, low quality 20-49%).

**Table 3:** Intraoperative variables.

Intraoperative variables	Study	Outcome	
		Piezosurgery group	Control group
INTRAOPERATIVE BLEEDING	Kocyigit et al. (26)	0 cases	3 cases
	Bharati et al. (24)	10 patients (50% n): complete hemorrhage control. 10 patients (50% n): intermittent hemorrhage control.	1 patient (5% n): complete hemorrhage control. 12 patients (60% n): intermittent hemorrhage control. 7 patients (35% n): no hemorrhage control.
EASE OF CUTTING	Yaman et al. (25)	-	Greater ease of cutting.
CYST EPITELIAL PERFORATION	Kocyigit et al. (26)	0 cases	5 cases
	Yaman et al. (25)	VAS: No significant differences	VAS: No significant differences
DIFFICULTY OF INTERVENTION	Kocyigit et al. (26)	0 cases	5 cases
	Yaman et al. (25)	VAS: Greater ease of intervention in groups treated with piezoelectric surgery.	-
SOFT TISSUE DAMAGE	Kocyigit et al. (26)	0 cases	0 cases
	Bharati et al. (24)	No significant differences	No significant differences
INTERVENTION TIME	Kocyigit et al. (26)	100 % time exceed	0% time exceed
	Marra et al. (28)	Average of 18 minutes	Average of 12 minutes
	Yaman et al. (25)	Simple surgery: 37,5 ± 9,5 minutes Complicated surgeries: 88,4 ± 28,1 minutes	Simple surgery: 28,9 ± 5,2 minutes Complicated surgeries: 63,4 ± 8,1 minutes

VAS=visual analog scale, n=sample size.

**Table 4:** Postoperative and long-term variables.

Postoperative variables	Study	Outcome	
		Piezosurgery group	Control group
HEMORRHAGE	Kocyigit et al. (26)	0 cases	2 cases
PAIN	Pappalardo et al. (27)	Days (VAS): 1º (4,2) 2º (3,17) 3º (2,15) 4º (0,98) 5ª (0,54) 6º (0,54) 7º (0,54)	Days (VAS): 1º (8,34) 2º (7,56) 3º (6,54) 4º (5,17) 5º (5,17) 6º (3,17) 7º (2,68)
	Bharati et al. (24)	VAS: Less postoperative 1 - 2 days. VAS: No difference until 7 days.	-
	Marra et al. (28)	24- 48- 72 h: P < C	24- 48- 72 h: P < C
INFECTION	Yaman et al. (25)	2 cases	2 cases
EDEMA	Pappalardo et al. (27)	24 h = 18 mm 48 h = 18 mm 72 h = 13mm 1 week =11 mm	24 h = 6 mm 48 h = 51 mm 72 h = 48 mm 1 week = 17 mm
	Marra et al. (28)	24h = 2,23 mm 48h = 1,34 mm 72h = 0,76 mm	24h = 5,82 mm 48h = 4,79 mm 72h = 2,84 mm
PARESTESIA	Pappalardo et al. (27)	0 cases	2 cases (8%)
DEHISCENCE	Yaman et al. (25)	2 cases (< 5 mm)	4 cases (< 5 mm)
RECURRENCE	Yaman et al. (25)	Odontogenic keratocyst N=5 Recurrences= 0	Odontogenic keratocyst N=4 Recurrences= 0
	Yaman et al. (25)	Dentigerous cyst N=6 Recurrences= 0	Dentigerous cyst N=6 Recurrences= 0
	Yaman et al. (25)	Radicular cyst N=32 Recurrences= 0	Radicular cyst N=29 Recurrences= 0
	Kocyigit et al. (26)	Radicular cyst N=19 Recurrences= 0	Radicular cyst N=10 Recurrences= 2

P=piezosurgery group, C= control group, VAS=visual analog scale.

## Data Availability

The datasets generated during and/or analyzed during the current study are available from the Correspondence on reasonable request.
